# The Many Guises of Endometriosis: Giant Abdominal Wall Endometriosis Masquerading as An Incisional Hernia

**DOI:** 10.22074/ijfs.2018.5126

**Published:** 2017-10-14

**Authors:** Chiara Petrosellini, Sala Abdalla, Tayo Oke

**Affiliations:** Department of General Surgery, Queen Elizabeth Hospital, London, United Kingdom

**Keywords:** Ascites, Endometriosis, Infertility, Laparotomy

## Abstract

Endometriosis is defined by the presence of ectopic endometrial tissue outside the uterine cavity. Although it is a leading cause of chronic pelvic pain and infertility, its clinical presentation can vary, resulting in diagnostic and therapeutic challenges. Extrapelvic endometriosis is particularly difficult to diagnose owing to its ability to mimic other conditions. Endometrial tissue in a surgical scar is uncommon and often misdiagnosed as a granuloma, abscess, or malignancy. Cyclical hemorrhagic ascites due to peritoneal endometriosis is exceptionally rare. We report the case of a pre-menopausal, nulliparous 44-year-old woman who presented with ascites and a large abdominal mass that arose from the site of a lower midline laparotomy scar. Five years previously, she had undergone open myomectomy for uterine fibroids. Soon after her initial operation she developed abdominal ascites, which necessitated percutaneous drainage on multiple occasions. We performed a laparotomy with excision of the abdominal wall mass through an inverted T incision. The extra-abdominal mass consisted of mixed cystic and solid components, and weighed 1.52 kg. It communicated with the abdominopelvic cavity through a 2 cm defect in the linea alba. The abdomen contained a large amount of odourless, brown fluid which drained into the mass. There was a large capsule that covered the small and large bowel, liver, gallbladder, and stomach. Final histology reported a 28×19×5 cm mass of endometrial tissue with no evidence of malignant transformation. The patient recovered well post-operatively and has remained asymptomatic. Our case illustrates that, despite being a common disease, endometriosis can masquerade as several other conditions and be missed or diagnosed late. Delay in diagnosis will not only prolong symptoms but can also compromise reproductive lifespan. It is therefore paramount that endometriosis is to be considered early in the management of premenopausal women who present with an irregular pelvic mass or hemorrhagic ascites.

## Introduction

Endometriosis is a histological diagnosis defined by the presence of endometrial glandular and stromal tissue outside the uterine cavity. It affects around 10% of women of reproductive age and is frequently implicated in infertility (1). It is rarely found before menarche and tends to regress after menopause. Although endometriosis is considered to be a benign condition, a well-established association exists between longstanding lesions and the development of clear cell and endometrioid carcinomas (2). The human endometrium is hormone-dependent and undergoes cyclical hyperplasia, secretion, and shedding. The ectopic endometrial foci in endometriosis respond to cyclical hormonal changes in the same way as the intrauterine endometrium, which results in focal bleeding, inflammation, and fibrosis. This manifests in symptoms which vary in frequency and intensity, including dysmenorrhea, menorrhagia, dyspareunia, and pelvic pain. 

The etiology and pathogenesis of endometriosis are not fully understood. Several proposed theories include retrograde menstruation first described by Sampson (3), coelomic metaplasia, and mestastatic spread. More recent research has proposed that altered immunity, stem cells, and epigenetic changes are implicated in the disease process (4,7). The common sites for ectopic foci of endometrial tissue are the ovaries, fallopian tubes, vagina, cervix, rectovaginal septum, and the uterosacral ligaments (8). Extrapelvic implantation in a number of organs such as the gastrointestinal (GI) tract, lungs, pleura, kidneys, bladder, and brain has been reported (8,9). The uterosacral ligaments and posterior cul-de-sac are the most common site of pelvic implantation while the GI tract is the most common extrapelvic site of endometriosis. Although endometriosis in a surgical scar is rare, there are several reports on endometriosis in Pfannenstiel incisions following cesarean sections (10,14). There is a paucity of literature that relates to abdominal wall endometriosis following other surgical procedures. 

We report the case of a pre-menopausal, nulliparous 44-year-old woman who presented with ascites and a large abdominal mass that arose from the site of a lower midline laparotomy scar. After extensive investigation, the final histology confirmed endometriosis as the cause of her unusual clinical presentation. 

## Case report

A 44-year-old Nigerian female was admitted with a large, symptomatic abdominal mass. She was para 0, gravida 0 and in 2011 underwent open myomectomy through a lower midline abdominal incision for large, symptomatic fibroids. She also had a background of pulmonary tuberculosis (TB) which was treated in 1995, iron-deficiency anemia, and a benign goiter. We received the consent of all patients. Within the first few months following her open myomectomy she began to develop abdominal ascites, which was drained percutaneously on approximately three occasions in 2011-12. Owing to some personal circumstances, she did not attend her regular outpatient appointments and was eventually lost to follow-up. However, her abdomen continued to swell, with expansion of the skin and subcutaneous tissue that surrounded the scar. 

She had no other relevant gynecological history and, aside from ferrous sulfate, she took no other regular medications. She was a non-smoker, denied alcohol use, and lived independently with her extended family. In 2016, five years following her original open myomectomy, she presented with a large abdominal mass that appeared to arise from the abdominal wall. This mass began to spontaneously discharge large volumes of brown fluid on the day of her admission. She was systemically well and gave no history of change in bowel habit or obstructive symptoms. She was not sexually active and had a normal menstrual history. She was not up-to-date with smear testing but denied intermenstrual bleeding. Her last menstrual period was one day previously. 

**Fig.1:**
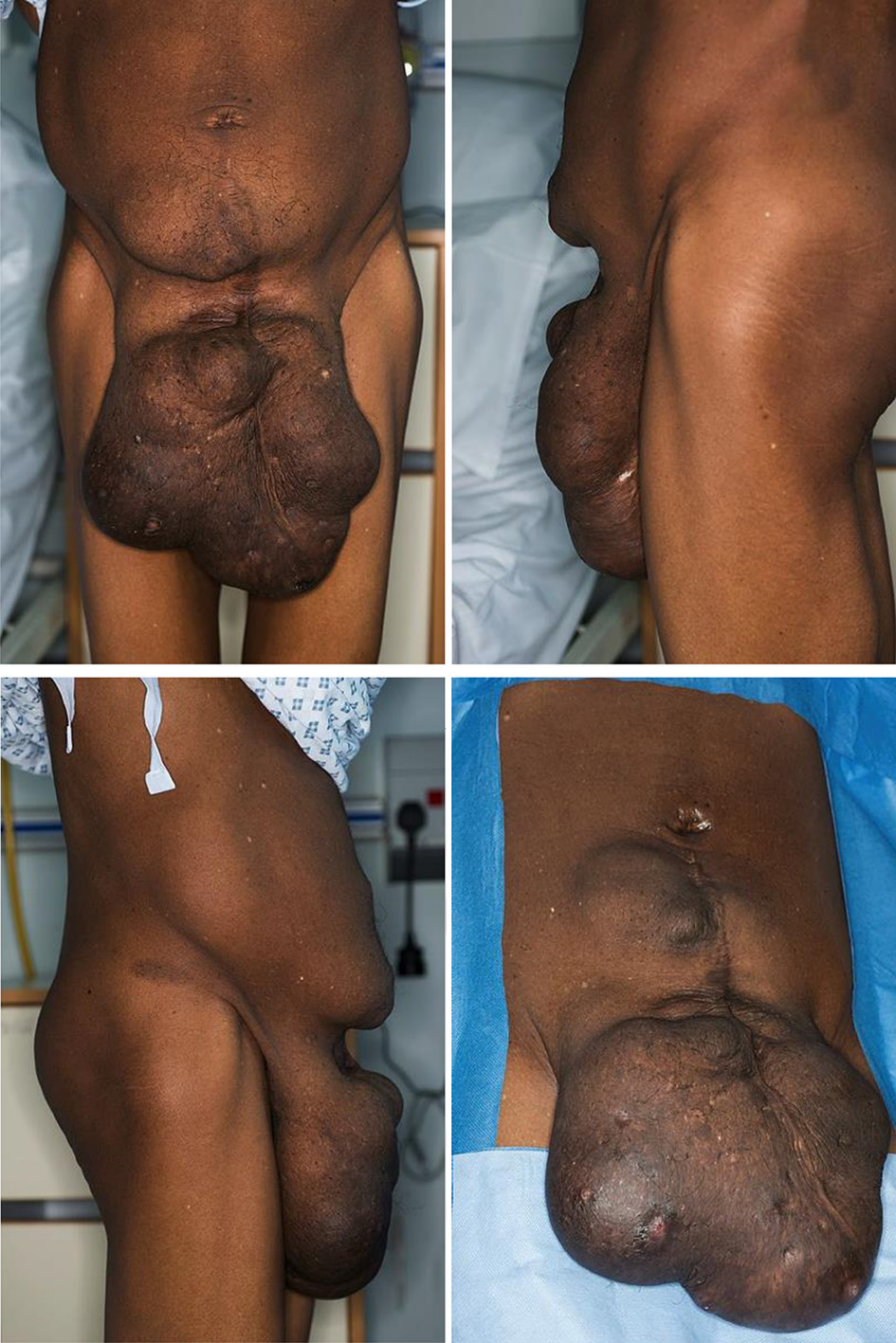
Pre-operative photographs of the multi-lobulated lesion with an overlying lower midline laparotomy scar from a previous open myomectomy. Multiple small puncti can be seen on the skin.

**Fig.2:**
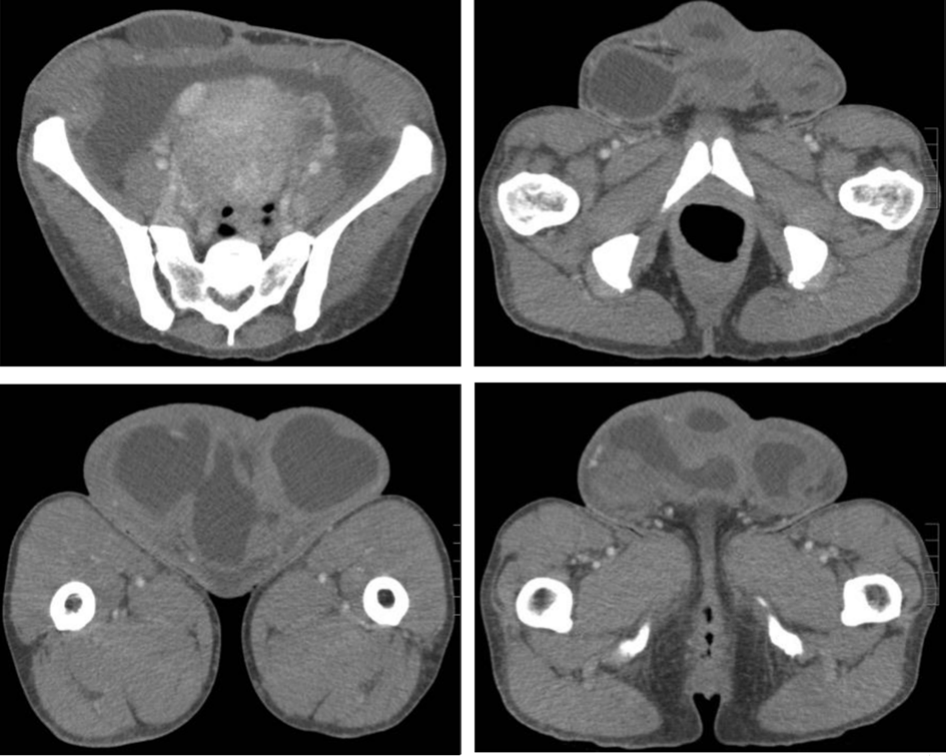
Axial sections from a computed tomography (CT) scan with intravenous contrast that demonstrated a large, extraperitoneal lobulated spaceoccupying lesion with mixed cystic and solid components.

On clinical examination she was thin, weighed 48 kg, with a body mass index (BMI) of 18. She was hemodynamically stable with a normal cardiorespiratory examination. She had a soft abdomen, with evidence of shifting dullness, and a large, irregular, firm mass that arose from the lower half of the abdomen overlain by her lower abdominal midline scar (Fig .1). It measured approximately 30×20×10 cm. The overlying skin was of variable thickness with several small puncti on its inferior aspect, one of which discharged brown, odorless fluid. The mass itself was non-tender. Digital rectal examination was unremarkable. Bowel sounds were normal and could not be detected on auscultation of the mass. The clinical impression was that this was a large incisional hernia with evidence of ascites. Laboratory blood results revealed a microcytic anemia (hemoglobin count 10.1 g/dL, mean cell volume 63.6 fL), normal white cell count, renal and liver functions, and a mildly elevated C-reactive protein of 33 mg/L. Abdominal and chest radiographs were unremarkable. A computed tomography (CT) scan of her abdomen and pelvis confirmed the presence of a large, extraperitoneal lobulated space-occupying lesion with a mixed cystic and solid appearance that extended at least 20 cm caudally (Fig .2). There was an 8×11×13 cm lesion in the uterus and a moderate amount of ascites, mainly in the lower abdomen and pelvis. 

**Fig.3:**
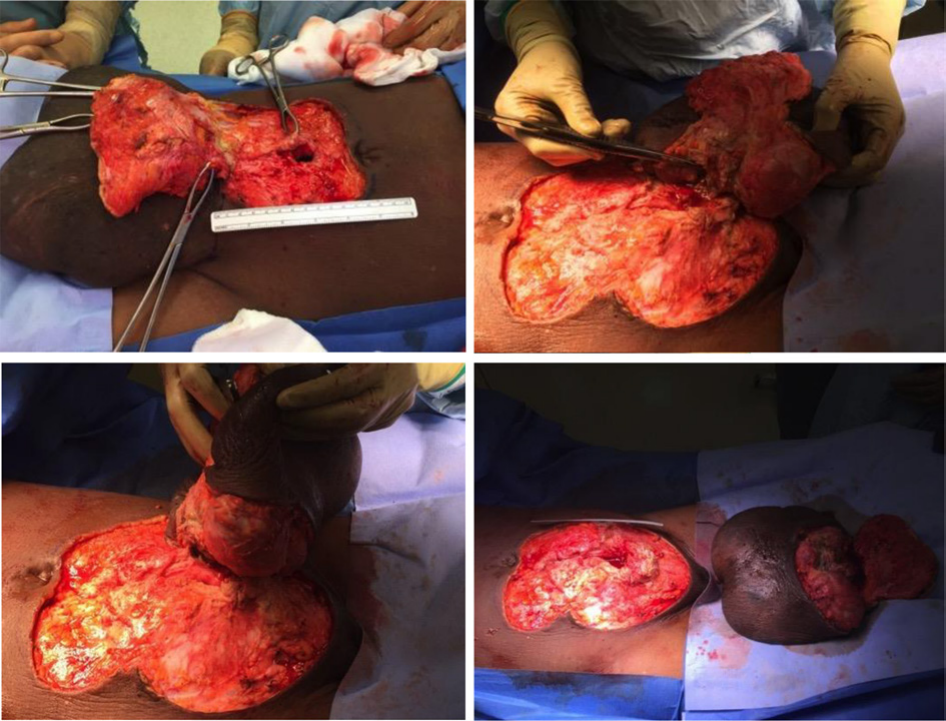
Findings at laparotomy; the extra-abdominal mass was of mixed cystic and solid components and communicated with the abdominopelvic cavity through a 2 cm defect in the linea alba just below the umbilicus. The specimen weighed 1.52 kg.

A follow-up pelvic ultrasound scan (USS) demonstrated a bulky uterus that measured 15×11.5×9.7 cm with gross pelvic ascites. The differential diagnosis was a possible uterine malignancy with peritoneal and abdominal wall carcinomatosis or disseminated TB, particularly in light of her previous pulmonary TB. Assessment of her tumor markers showed that both the CEA and CA19-9 were within normal range; CA 125 was elevated at 89.8 U/mL (0-35 U/mL). Her hepatitis (A, B, C, E), human immunodeficiency virus (HIV), autoimmune serology and sickle cell tests were all negative. She underwent ultrasound guided peritoneal biopsy for TB screening, the histology of which showed chronic inflammation with no evidence of granulomatous inflammation or malignancy. Additionally, her ascitic fluid was negative for Mycobacterium tuberculosis. Two weeks following her original admission she underwent a laparotomy with excision of the large abdominal wall mass. This was carried out through an inverted T incision that encircled the lower midline laparotomy incision and the mass, but preserved the umbilicus. The extra-abdominal mass was of mixed cystic and solid components, and weighed 1.52 kg. It communicated with the abdominopelvic cavity through a 2 cm defect in the linea alba just below the umbilicus. The abdominopelvic cavity contained a large amount of odorless, brown fluid which flowed into the mass. There was an enlarged uterus bulky with fibroids. There were two large endometrioid cysts bilaterally in the region of the adnexae. The ovaries could not be clearly delineated and the fallopian tubes adhered to the pelvic walls. There was a large capsule that covered the small and large bowel, liver, gallbladder, and stomach. The mass was dissected free from the abdominal wall fascia and excised (Fig .3). The endometrioid cysts that contained altered blood were drained. The large abdominopelvic capsule was stripped off of its adherent viscera, but it was not safe to strip it off in its entirety. 

**Fig.4:**
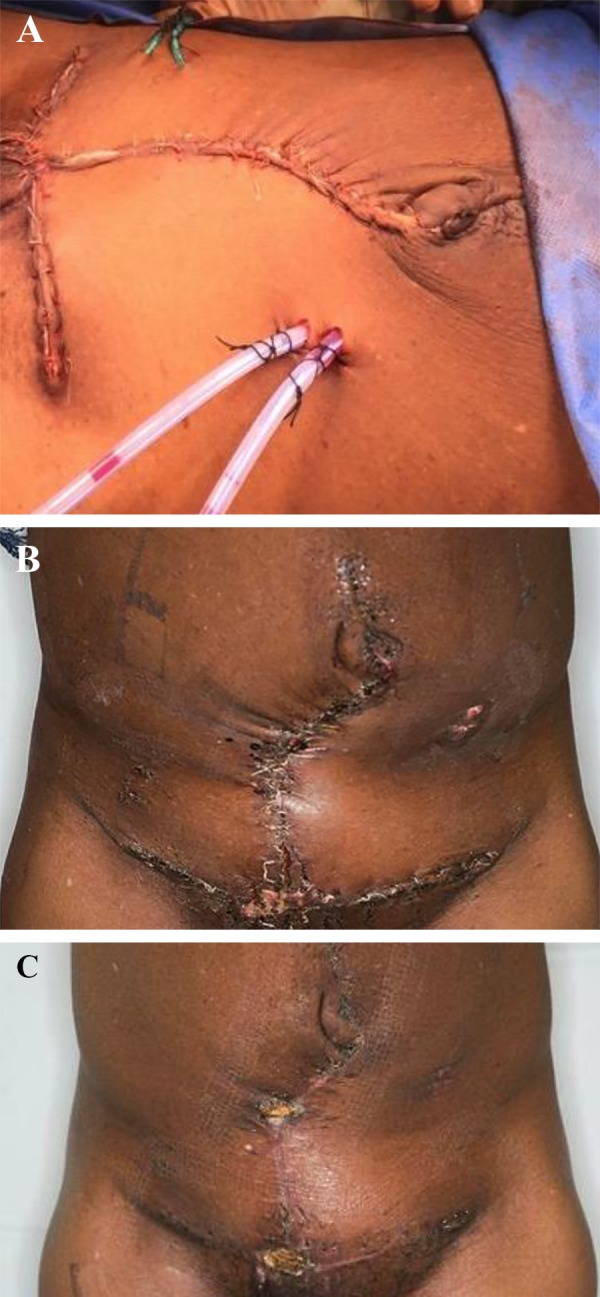
The skin was closed with horizontal mattress sutures using 2/0 Vicryl Rapide. A. The umbilicus was preserved. Two large Robinson drains were left in the abdominopelvic cavity, and two negative pressure (Redivac) drains were left in the subcutaneous space, B. Two weeks postoperative after all the drains had been removed, and C. Five weeks postoperative.

The fascial opening, which extended caudally by 5 cm, was closed with interrupted 1 Nylon sutures. The umbilicus was preserved. We left two large Robinson drains in the abdominopelvic cavity and two negative pressure (Redivac) drains remained in the subcutaneous space. The skin was closed with horizontal mattress sutures using 2-0 Vicryl Rapide. A negative pressure incision management system (PICO dressing) was applied to the wound. The final histology confirmed a 28×19×5 cm mass of endometrial tissue with no evidence of malignant transformation. The patient made a good post-operative recovery and was discharged one week following surgery with the abdominal drains and planned gynecological follow-up. She was assessed on a weekly basis in the General Surgery Outpatient Department in general surgery outpatients. All drains were removed 10 days following discharge (Fig .4). The patient provided written informed consent for publication of this case report and accompanying images. She remains well. 

## Discussion

Several theories have been proposed to explain the pathogenesis of ectopic endometrial tissue (15). Endometriosis was first described by Rokitansky in 1860. Adenomyosisthe presence of endometrial tissue within the myometriumwas then described in more detail by the pathologist, Iwanoff, and the surgeon, Cullen. They believed that metaplasia of embryonic cells caused the ectopic endometrial tissue (16). The theory of coelomic metaplasia has remained as one of the leading models of endometriosis. It is based on the idea that peritoneal cells can differentiate into endometrial cells following chronic inflammatory stimulus (17). 

Another leading theory is retrograde menstruation. In 1927, Sampson published his work on peritoneal and ovarian endometriomas. He proposed that reversed menstrual flow through the fallopian tubes led to implantation of endometrial cells into the peritoneal cavity, with subsequent endometriosis (3). This explanation has been debated, however, as it has not explained pre-pubertal endometriosis. The presence of ectopic endometrial tissue has even been identified in human female fetuses of different gestational ages (18). Sampson’s theory does not explain the similar incidence of retrograde menstruation in women with and without endometriosis (19). 

The metastatic theory is often favored, although it has never been scientifically demonstrated. This theory suggests that endometrial tissue can be transported to adjacent locations via surgical intervention, hematogenous or lymphatic spread. This can explain the presence of endometriosis in very distant sites such as the pleura and brain. Increasing evidence exists for the role of epigenetics, oxidative stress, and immune dysfunction in the growth of ectopic endometrial tissue (20). 

The clinical presentation of endometriosis is extremely variable and this leads to diagnostic and therapeutic challenges. Extrapelvic endometriosis is particularly difficult to diagnose due to its wide spectrum of presentations and ability to mimic several other conditions. Our patient presented to the general surgeons with an irregular abdominal mass that resembled an incisional hernia, but with associated weight loss and microcytic anemia. In view of her previous history of pulmonary TB, the two main differentials in her diagnosis at this stage were a gastro-intestinal malignancy and intra-abdominal TB. A transvaginal/transabdominal USS demonstrated an irregular, bulky uterus, and ascites. Uterine malignancy was then included in the differential diagnosis. It was only at laparotomy that the likely diagnosis of endometriosis became apparent. The abdominal wall protrusion that arose from her lower midline laparotomy scar and mimicked an incisional hernia, was confirmed as giant endometriosis. We identified two large ovarian endometriomas in the region of the adnexae, which confirmed concurrent pelvic endometriosis. The abdomen and pelvis were not explored in more detail at the time of her surgery, however the finding of bilateral ovarian endometriomas was highly suggestive of extensive pelvic endometriosis with a high chance of bowel involvement. 

Endometriosis in a surgical scar is a rare entity, with an incidence of 0.03 to 0.15%. It is often misdiagnosed as a granuloma, abscess, or malignancy (10,21,22). We have found a few cases in the literature of abdominal wall endometriosis after surgical procedures. In a review of 445 cases of abdominal wall endometriosis, 57% occurred following cesarean sections, 11% after hysterectomy, and 12% have followed all other abdominal surgeries (23). Up to 80% of patients complain of scar pain which is often cyclical in nature. Interestingly, however, our case demonstrates that pain in cutaneous endometriosis is not always present. This patient’s case supports the paradox often observed in endometriosis; small lesions can often be very painful, whereas marked disease is often not painful (24). Incisional endometriosis may, in part, be consistent with the metastatic theory of endometriosis, as endometrial tissue may displace to the wound during pelvic surgery. It could also be explained by metaplasia of stem cells during the healing process. It was unclear whether the uterine cavity was breeched during the original myomectomy that our patient underwent. The case we have described is most likely the result of metaplasia of undifferentiated cells located in the abdominal wall during the healing process following myomectomy. 

Recurrent hemorrhagic ascites due to peritoneal or pelvic endometriosis is also rare; less than 50 cases have been described in the literature (25,26). Endometrial tissue is highly dynamic and responds to cyclical hormonal changes. The development of hemorrhagic ascites in endometriosis is cyclical and most symptomatic during menstruation. Our patient sought medical attention because large volumes of brown fluid spontaneously discharged from her abdominal wall earlier that day. This had coincided with the onset of her menstrual period which was the day prior to this presentation. Pelvic TB and malignancy are common causes of hemorrhagic ascites (27,28). Disseminated TB, in particular, was an important differential in the case of our patient in view of her previous history of pulmonary TB. However, endometriosis should always be considered in nulliparous women of childbearing age who present with hemorrhagic ascites. 

Our patient received surgical treatment five years after the onset of her symptoms. Her complex social situation and failure to attend regular follow-up led to a delay in her diagnosis. Once extrapelvic endometriosis has been identified, surgical treatment appears to result in a cure in over 95% of cases (23). Following surgery, progressive reduction in hemorrhagic ascites has been observed, with complete remission within six months (27). The patient has recovered well post-operatively. At present, she remains asymptomatic. Her extensive pelvic endometriosis, however, was not treated at the time of laparotomy and excision of the abdominal wall mass. She is under follow-up by the gynecology team with the intent for further surgery. 

## Conclusion

Our case illustrates that, despite being a common disease, endometriosis can masquerade as several other conditions; therefore, it can be missed or diagnosed late. Delay in diagnosis will not only prolong symptoms but can also compromise reproductive lifespan. It is therefore crucial that endometriosis is considered early in the management of premenopausal women who present with an irregular pelvic mass or hemorrhagic ascites. 

## References

[1] Wheeler JM (1989). Epidemiology of endometriosis-associated infertility. J Reprod Med.

[2] Krawczyk N, Banys-Paluchowski M, Schmidt D, Ulrich U, Fehm T (2016). Endometriosis-associated malignancy. Geburtshilfe Frauenheilkd.

[3] Sampson JA (1927). Peritoneal endometriosis due to the menstrual dissemination of endometrial tissue into the peritoneal cavity. Am J Obstet Gynecol.

[4] Králíčková M, Vetvicka V (2015). Immunological aspects of endometriosis: a review. Ann Transl Med.

[5] Du H, Taylor HS (2007). Contribution of bone marrow-derived stem cells to endometrium and endometriosis. Stem Cells.

[6] Zanatta A, Rocha AM, Carvalho FM, Pereira RM, Taylor HS, Motta EL (2010). The role of the Hoxa10/HOXA10 gene in the etiology of endometriosis and its related infertility: a review. J Assist Reprod Genet.

[7] Lee B, Du H, Taylor HS (2009). Experimental murine endometriosis induces DNA methylation and altered gene expression in eutopic endometrium. Biol Reprod.

[8] Jubanyik KJ, Comite F (1997). Extrapelvic endometriosis. Obstet Gynecol Clin North Am.

[9] Markham SM, Carpenter SE, Rock JA (1989). Extrapelvic endometriosis. Obstet Gynecol Clin North Am.

[10] Jubanyik KJ, Comite F (1997). Extrapelvic endometriosis. Obstet Gynecol Clin North Am.

[11] Pathan ZA, Dinesh US, Rao R (2010). Scar endometriosis. J Cytol.

[12] Brenner C, Wohlgemuth S (1990). Scar endometriosis. Surg Gynaecol Obstet.

[13] Daye SS, Barone JE, Lincer RM, Blabey RC, Smego DR (1993). Pfannenstiel Syndrome. Am Surg.

[14] Gajjar KB, Mahendru AA, Khaled MA (2008). Caeserean scar endometriosis presenting as an acute abdomen: a case report and review of the literature. Arch Gynaecol Obstet.

[15] Sourial S, Tempest N, Hapangama DK (2014). Theories on the pathogenesis of endometriosis. Int J Reprod Med.

[16] Benagiano G, Brosens I (2011). Who identified endometriosis?. Fertil Steril.

[17] Ferguson BR, Bennington JL, Haber SL (1969). Histochemistry of mucosubstances and histology of mixed müllerian pelvic lymph node glandular inclusions.Evidence for histogenesis by müllerian metaplasia of coelomic epithelium. Obstet Gynecol.

[18] Signorile PG, Baldi F, Bussani R, Viceconte R, Bulxomi P, D’Armiento M (2012). Embryonic origin of endomtriosis: analysis of 101 human female foetuses. J Cell Physiol.

[19] Halme J, Hammond MG, Hulka JF, Raj SG, Talbert LM (1984). Retrograde menstruation in healthy women and in patients with endometriosis. Obstet Gynecol.

[20] Forte A, Cipollaro M, Galderisi U (2014). Genetic, epigenetic and stem cell alterations in endometriosis: new insights and potential therapuetic perspectives. Clin Sci (Lond).

[21] Francica G, Giardiello C, Angelone G, Cristiano S, Finelli R, Tramontano G (2003). Abdominal wall endometriomas near cesarean delivery scars: sonographic and color doppler findings in a series of 12 patients. J Ultrasound Med.

[22] Wolf GC, Singh KB (1989). Cesarean scar endometriosis: a review. Obstet Gynecol Surv.

[23] Horton JD, Dezee KJ, Ahnfeldt EP, Wagner M (2008). Abdominal wall endometriosis: a surgeon’s perspective and review of 445 cases. Am J Surg.

[24] Demco L (1998). Mapping the source and character of pain due to endometriosis by patient-assisted laparoscopy. J Am Assoc Gynecol Laparosc.

[25] Ekoukou D, Guilherme R, Desligneres S, Rotten D (2005). Endometriosis with massive hemorrhagic ascites: a case report and review of the literature. J Gynecol Obstet Biol Reprod (Paris).

[26] Lin JN, Lin HL, Huang CK, Lai CH, Chung HC, Liang SH (2010). Endometriosis presenting as bloody ascites and shock. J Emerg Med.

[27] Shabeerali TU, Rajan R, Kuruvilla AP, Noronha S, Krishnadas D, Shenoy KT (2012). Hemorrhagic ascites: are we missing endometriosis?. Indian J Gastroenterol.

[28] Ertas IE, Gungorduk K, Ozdemir A, Emirdar V, Gokcu M, Dogan A (2014). Pelvic tuberculosis, echinococcosis, and actinomycosis: great imitators of ovarian cancer. Aust N Z J Obstet Gynaecol.

